# Medication Adherence among Patients with Diabetes Mellitus and Its Related Factors—A Real-World Pilot Study in Bulgaria

**DOI:** 10.3390/medicina59071205

**Published:** 2023-06-26

**Authors:** Rayana Dinkova, Lyubomir Marinov, Miglena Doneva, Maria Kamusheva

**Affiliations:** 1 Department of Organization and Economics of Pharmacy, Faculty of Pharmacy, Medical University of Sofia, 1000 Sofia, Bulgaria; rayana_dinkova@abv.bg (R.D.); mdoneva@pharmfac.mu-sofia.bg (M.D.); 2 Department of Pharmacology, Pharmacotherapy and Toxicology, Faculty of Pharmacy, Medical University of Sofia, 1000 Sofia, Bulgaria; lmarinov@pharmfac.mu-sofia.bg

**Keywords:** diabetes, medication adherence, diabetes management, quality of life

## Abstract

*Background and Objectives*: The objective is to evaluate medication adherence level (MA) and the relevant determinants of MA among patients with type 2 diabetes mellitus (T2DM) monitored in ambulatory settings by general practitioners. *Materials and Methods*: A cross-sectional study was conducted among patients with T2DM monitored in a GP practice in Sofia, Bulgaria (September–December 2022). All patients were interviewed according to a predesigned questionnaire after granting informed consent. MA level was evaluated through the Morisky–Green four-item questionnaire, and health-related quality of life was evaluated by EQ-5D-5L and VAS (visual analogue scale). Data were aggregated and statistically evaluated. *Results*: The total number of observed patients was 93. Around 48.4% of patients were female, and 90.3% of patients were between 50 and 80 years of age. Multimorbidity was identified among 70% (*n* = 65) of the respondents. High and medium levels of MA were revealed in 64.51% and 33.3% of respondents, respectively. Patients treated with insulin secretagogues were most adherent to the therapy (*n* = 83.3%) in comparison with the other treatment groups. The onset of the disease, professional status, age, gender, number of therapies, and quality of life did not affect the level of MA (*p* > 0.05). VAS scores among nonsmokers (VAS = 63.16 ± 20.45 vs. 72.77 ± 14.3) and non-consumers of alcohol (VAS = 63.91 ± 19.34 vs. VAS = 72.54 ± 15.98) were statistically significant lower (*p* < 0.05). A significant related factor for MA was years lived with diabetes (OR = 3.039, 95% CI 1.1436–8.0759, *p* = 0.0258). The longer the disease duration, the more the odds for a high MA level increased. *Conclusions*: The number of nonadherent diabetic patients in Bulgaria is low, which might be evidence of patients’ concern about their own health and understanding about the importance of prescribed therapy. Further comprehensive study with additional patients is required to confirm the results and investigate the predicting factors for a high level of MA.

## 1. Introduction

Diabetes mellitus is a metabolic disorder characterized by hyperglycemia due to impaired insulin secretion, impaired insulin action, or both. Early detection of diabetes and control of risk factors, including control of blood sugar, blood pressure, duration of the disease, and control of blood lipids, is of utmost importance for the development and severity of the disease [1]. Diabetes is a chronic disease that requires long-term care and patients’ education for the purposes of prevention of complications [2]. The number of Bulgarian citizens with diabetes mellitus in the outpatient lists for 2018 was 503,753. The number of newly diagnosed patients with diabetes mellitus for the analyzed period was 55,172. The number of deceased patients with diabetes mellitus for the analyzed period was 26,813; 18,641 of them had a physician visit in 2018 [3]. The economic burden of the disease is forecast to grow from USD 1.3 trillion in 2015 to USD 2.1 trillion under by 2030 [4]. According to data provided in the literature, 4.4 million people worldwide are affected by the disease [5].

Adherence has been defined as “the extent to which a person’s behavior (in terms of taking medications, following diets, or executing other lifestyle changes) coincides with the agreed recommendations from a health care provider” [6]. It is dependent upon a variety of factors such as patients’ characteristics, the patient and their family members’ behavior, interactions with healthcare providers, and the healthcare system itself [7]. Medication nonadherence is defined as “taking less than 80% of prescribed doses”. However, nonadherence could be related with taking too many doses. Nonadherence is associated with a high risk for poor health status, mortality, and complications [8].

The key to achieving the desired therapeutic outcomes is a high level of adherence. Diabetes has serious long-term consequences on health and requires early and timely medical care and preventive measures. Enhanced glycemic control, which can be achieved through a high level of medication adherence, can significantly reduce microangiopathic and macroangiopathic complications [9]. Studies have shown that patients treated with fixed doses have lower variations in their number of prescriptions. Therefore, these patients have a higher level of change in their medication adherence and increased persistence in comparison with patients treated with loose-dose combinations. Results show that a fixed-dose combination for treatment of type 2 diabetes is related to improvement in medication adherence levels in comparison with a loose-dose combination [10,11].

A study has investigated factors influencing the level of adherence among patients with diabetes [11]. Some of the factors that decrease medication adherence are beliefs and lifestyle modifications. These have a larger impact on diabetes control than medications. Reasons for medication nonadherence could be medication costs and poor communication with providers. The belief that diabetes medication is important to maintain a good health status, and the presence of family support and providers are some of the main factors for medication adherence improvement [11]. Measures to improve patient satisfaction and medication adherence levels, such as simplifying the prescribing regimen, educational programs, improved communication between patients and healthcare professionals, reminders, and lower treatment costs, must be multifactorial [8].

The main goal of the current survey is to evaluate the medication adherence (MA) level and the relevant determinants of MA among individuals with type 2 diabetes mellitus (T2DM) followed up by general practitioners. The other crucial aim was to measure the patients’ quality of life and its correlation with MA level.

## 2. Materials and Methods

### 2.1. Design, Setting, Patient Recruitment, and Sample Size

We conducted a cross-sectional, 4-month study among 93 (7.13% of the total number of patients) of 107 patients with diabetes mellitus followed up by a general practitioner with 1304 total patients in Sofia city. All adult patients with type 2 diabetes over 18 years of age who were on pharmacotherapy (ICD codes E10, E11) and granted informed consent were included. The study period was between September and December 2022. Patients who were able to speak and understand Bulgarian language, had type 2 diabetes diagnosed at least one year before the study, visited GP office on site, and were caregiver-independent were eligible for the current study. Exclusion criteria included refusal to grant informed consent and persistence of any condition that could interfere with the patients’ ability to complete the questionnaire.

### 2.2. Characteristics and Cost of Therapy

Clinical data for each patient was collected on the basis of a specifically designed questionnaire. Demographic characteristics (age, gender), medical history of diseases, disease-specific data (type of diabetes mellitus, duration of the disease), pharmacotherapy, concomitant diseases, annual hospitalization rate, consumed medicines, risk factors, level of medication adherence, assessment of patients’ lifestyle, and days off work due to diabetes were collected. Healthcare costs were calculated for the 4-month period (September–December 2022) using micro-costing approach: direct costs (for diabetes treatment, hospitalization, and medications per year) and indirect costs (time off from work in days). Reimbursement levels were recorded for medicinal products for patients with diabetes for those part of the Positive Drug List (PDL). Each product was recorded using the unit price covered by the NHIF (National Health Insurance Fund), a single-payer healthcare system administering the compulsory health insurance in the country (the rate of coverage is 8% for 2023, including an employee contribution of 3.2%) [12]. The PDL only consists of prescription paid for by the NHIF, the Ministry of Health, and the hospitals’ budgets [12].

The indirect costs were calculated using the number of days spent in hospital by every patient (extracted from the questionnaire completed by each patient) and gross domestic product per capita for 2022 (source of data: National Statistical Institute, Bulgaria [13]). This type of indirect costs was calculated on the basis of days out of work (absenteeism) data, applying the human capital approach formula.

The number of patients was multiplied by the number of days out of work for the observed period and by the average salary per capita per day. Indirect costs were represented as lost productivity using the following formula for the human capital approach:

Indirect cost = n × d × p/N

n—number of hospitalized patients (12)
d—number of days out of work (5)
p—gross domestic product per capita in Bulgaria for 2022 (BGN 18,996.00)
N—number of working days in 2022 (248)

On the basis of the responses collected, 12 patients experienced loss of productivity (BGN 4595.80). Assuming they all earned the average wage for the country, BGN 1583.00, this would amount to BGN 18,996.00 for 1 year [13]. The number of working days for 2022 was 248.

The collected data, such as demographic and clinical data, quality of life and economic outcomes, were considered in order to identify independent variables of nonadherence among the observed patients with diabetes.

### 2.3. Evaluation of Medication Adherence and Quality of Life

We used the definition for MA provided by ABC project (Ascertaining Barriers to Compliance: policies for safe, effective and cost-effective use of medicines in Europe). “Adherence to medications” is defined as the process by which patients take the medicines as they were prescribed and recommended by their healthcare professional. It consists of three crucial phases: “Initiation”, “Implementation”, and “Discontinuation”.

The Morisky–Green 4-item questionnaire (medication adherence (MA) questionnaire) was applied in order to define MA level. It consists of 4 questions; each has two possible options (“yes” or “no”), as the range is between 0 and 4. The levels of MA are as follows: high (in case of 0 points), medium (1–2 points), and low (3–4 points) MA [14]. The questionnaire was administered to those participants with diabetes willing to be involved in the study.

Quality of life (QoL) was assessed using EQ-5D-5L (5-level EuroQol 5D version). This questionnaire is a unidirectional QoL measure providing utility value in the range of 0 (equal to death) and 1 (equal to perfect health). The questionnaire consists of 5 questions, each related to different aspect of quality of life (mobility, self-care, depression/anxiety, usual activities, pain/discomfort). Each dimension has 5 options for answering: no problems, slight, moderate, severe, and extreme problems. The answers provided are used to calculate a single index “utility” score through a specific algorithm. The UK value set and scoring algorithm are used to calculate the scores for each patient because a Bulgarian scoring algorithm is not available. The EQ-5D-5L includes a visual analogue scale which provides a single rating of self-perceived health between 0 and 100, representing “the worst health you can imagine” and “the best health you can imagine”, respectively [15].

The patients were asked to answer these questionnaires only once during the observation period: at the moment when they were invited for monitoring in the GP’s office.

### 2.4. Statistics

Appropriate statistical methods (descriptive statistics and comparison of proportions) were considered for the purposes of description and assessment of the correlations among the collected data. Through the method of descriptive statistics, we systematized the patients’ demographic characteristics including sex, age, place of birth, time of diagnosis, pharmacotherapy, quality of life, level of MA, etc. A comparison between the collected data for the patients was performed by MedCalc statistical software version 16.4.1. The factors influencing medication adherence levels, such as clinical, demographic data, quality of life, and cost variables, were evaluated. Logistic regression, considering the influencing factors, was estimated (*p* < 0.05 means statistical significance). The odds ratio (OR) of possessing high or low/medium level of MA was calculated using the method of logistic regression for several patient characteristics (gender, number of medicines prescribed (polypharmacy was defined as taking 5 or more medicines), multimorbidity (3 or more diseases), years lived with diabetes, and age).

### 2.5. Ethics

The study included all patients registered with the GP, with diabetes type 2, who visited the GP office for periodic health examinations and agreed to be involved in the study. These patients provided signed written informed consent, authorizing the investigators to use their anonymized (pseudonymized) data only for the purposes of the current study. The study was carried out in accordance with the requirements of the Declaration of Helsinki.

## 3. Results

### 3.1. Demographic Data and Risk Factors

The patients with diabetes admitted for periodic examination at the GP office (*n* = 107) during September–December 2022 were invited to answer the questions. Of the total, 93 patients agreed to participate in the study. The number of men and women participants was almost equal (45 men vs. 48 women). Most participants (90.33%) were in the age group over 50. Most participants were of retirement age (58.06%) and suffered from type 2 diabetes (98.92%). In 38.71% of the patients, the disease had been diagnosed within the last 5 years (Table 1). Of the 93 participants, 30 reported hospitalizations in the past year. These hospitalized patients were exposed to risk factors such as smoking (15 of the participants), use of alcoholic beverages (11 of the participants), and inability to maintain blood pressure within normal limits (10 of the participants).

### 3.2. Pharmacotherapy for Diabetes

**Figure 1:**
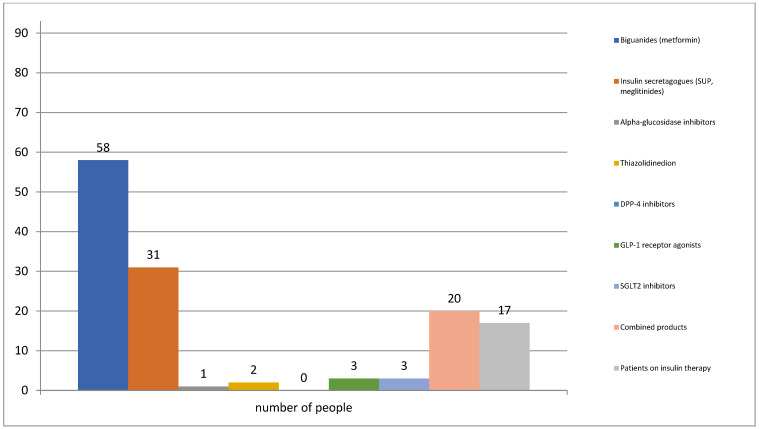
Prescribed pharmacotherapy for diabetes management among the observed group of patients.

Various pharmacotherapeutic groups have been used for the treatment of diabetes mellitus: biguanides, insulin secretagogues, thiazolidinediones, alpha-glucosidase inhibitors, DPP-4 inhibitors, GLP-1 receptor agonists, SGLT2 inhibitors, insulins, combined medicinal products (DPP4 inhibitors plus metformin ((sitagliptin; vildagliptin)/metformin), SGLT2 inhibitors plus metformin ((dapagliflozin; empagliflozin)/metformin)). Of the total, 58 of the surveyed patients were placed on treatment with biguanides (metformin) followed by treatment with insulin secretagogues (gliclazide MR, glimepiride) (*n* = 31). A significant number of patients were treated with combination products (*n* = 20) [Figure 1].

Most of the observed patients were diagnosed with non-insulin-dependent diabetes mellitus without complications (*n* = 73), followed by non-insulin-dependent diabetes mellitus with neurological complications (*n* = 16), and non-insulin-dependent diabetes mellitus with peripheral vascular complications (*n* = 4).

### 3.3. Pharmacotherapy for Concomitant Diseases

The definition of multimorbidity involves two or more medical diseases/conditions, each lasting over one year. Of the total number of participants, 72% were diagnosed with two or more additional chronic conditions and 28% were diagnosed with one additional chronic condition. Patients with this disease were more likely to die at an earlier age and to be hospitalized more often. Polypharmacy was defined as the routine use of five or more medications. Of the total, 53% of patients were using at least five or more medications. Of the total, 40% of patients were using monotherapy and 60% were using combined therapy.

The observed patients were diagnosed with the following diseases/conditions: autoimmune thyroiditis (*n* = 3), hypertensive heart with (congestive) heart failure (*n* = 1), hypertensive heart without (congestive) heart failure (*n* = 26), other types of angina pectoris (*n* = 10), atrial fibrillation and flutter (*n* = 9), heart failure (*n* = 9), consequences of cerebral infarction (*n* = 7), Parkinson’s disease (*n* = 2), primary open-angle glaucoma (*n* = 1), diabetic polyneuropathy (*n* = 14), etc.

The most common concomitant disease among the studied patient group was arterial hypertension (67.89%). Agents affecting the sympathoadrenal system were the most often prescribed antihypertensive therapy (*n* = 66), followed by combination products (*n* = 44) (Table 2).

Some of the patients were diagnosed with angina pectoris (*n* = 30), and all the respondents used statins. The most common diabetes complication among the observed patients was diabetic polyneuropathy (*n* = 14) as the preferred prescribed therapy included tioctic acid and uridine monophosphate.

### 3.4. Costs for Therapy

#### 3.4.1. Direct and Indirect Costs

**Figure 2:**
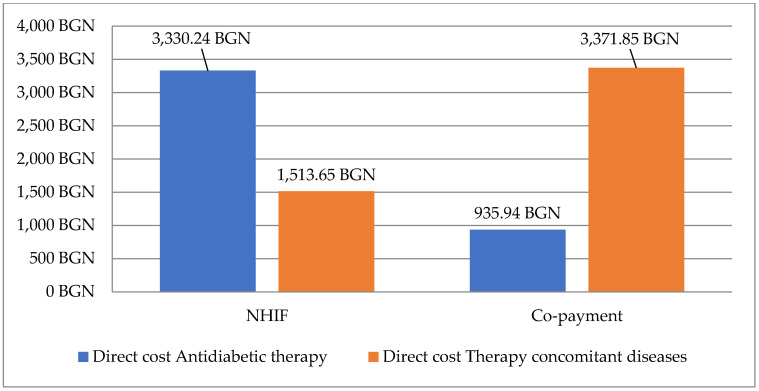
Direct cost for pharmacotherapy paid by NHIF and the observed patients.

Total direct monthly costs paid by NHIF were higher for antidiabetic therapy (BGN 3330.24 in comparison with BGN 935.94 for therapy for concomitant diseases) [Figure 2]. The total out-of-pocket payment was lower for patients on antidiabetic therapy in comparison with copayment for concomitant therapy (BGN 1513.65 BGN vs. BGN 3371.85).

**Figure 3:**
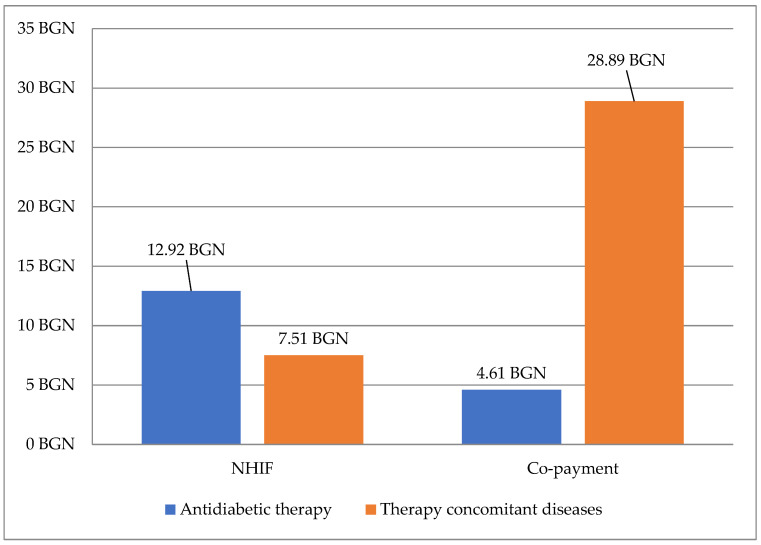
Median cost for diabetes therapy and for concomitant diseases by NHIF and the observed patients.

The median costs per patient per month paid by the fund were higher for the antidiabetic therapy (BGN 12.92) in comparison with the therapy for concomitant diseases (BGN 4.61) [Figure 3]. Out-of-pocket payment per patient was lower for antidiabetic therapy in contrast to concomitant therapy (BGN 7.51 vs. BGN 28.89).

We estimate that 12 patients will experience loss of productivity (BGN 4595.80 per patient).

#### 3.4.2. Total Cost

**Figure 4:**
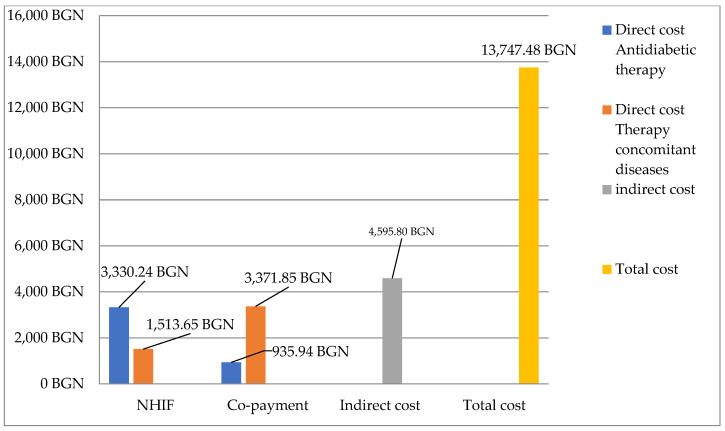
Monthly total costs for all observed patients paid by NHIF and patients.

The total cost for a month (public funds and patients) for all observed patients accounted for BGN 13 747.48. The costs are shown in Figure 4.

### 3.5. Quality of Life

The same number of patients completed both questionnaires (for QoL and for MA). On a VAS visual analog scale, patients provided a score ranging between 25 and 96 out of 100 units. When evaluated by EQ5D, the values ranged between −0.157 and 1.0, with the highest possible score being 1.

EQ-5D scores among nonsmokers (EQ-5D = 0.51 ± 0.32 vs. EQ-5D = 0.62 ± 0.31) (*p* > 0.05) and non-consumers of alcohol (EQ-5D = 0.49 ± 0.32 vs. EQ-5D = 0.648 ± 0.31) (*p* < 0.05) were lower than those for smokers and consumers of alcohol, respectively. Similar results for VAS scores (63.16 ± 20.45 vs. 72.77 ± 14.3 and VAS = 63.91 ± 19.34 vs. VAS = 72.54 ± 15.98, respectively (*p* < 0.05), were found (Table 3).

### 3.6. Level of Medication Adherence and Factors Influencing Medication Adherence 

The absolute number of respondents to the MA questionnaire was 86.9% (*n* = 93). High and medium levels of MA were revealed in 64.51% and 33.3% of respondents, respectively. Duration of the disease, professional status, age, gender, number of therapies, and quality of life did not affect the level of MA (*p* > 0.05). Those of the patients treated with monotherapy were more likely to comply with their treatment and demonstrate a higher level of adherence (36 vs. 21, *p* = 0.89) compared to those on dual therapy and those on triple therapy (36 vs. 3, *p* = 0.22).

When examining the factor “number of comorbidities” versus medication adherence, it was observed that most of the patients were in the ”high adherent” group regardless of the number of comorbidities. Among the group of highly adherent patients, the ratio of high adherents with three comorbidities was the highest. Comparison of the male and female groups did not show a significant difference in adherence levels (28 vs. 32, *p* = 0.78). In the female group, there was a higher propensity for high adherence compared to medium and low adherence. Comparing the working and retired groups, higher adherence was observed in the retired group (39 vs. 21, *p* = 0.069).

No statistically significant difference between the costs paid by the patients with a medium and high level of adherence was observed (BGN 32.89 ± BGN 23.85 vs. 37.31 ± 25.56, *p* = 0.43). Patients treated with insulin secretagogues were most adherent to the therapy (*n* = 83.3%) in comparison with the other treatment groups. In total, 17 out of 30 (56.67%) participants treated with metformin, and half of those on a combination therapy (metformin + insulin secretagogues), were high-adherent (Table 4).

A high level of MA was found among more women than men (OR = 1.7582, *p* = 0.2) as well as among those using polypharmacy (OR = 1.32 *p* = 0.5267); however, these differences were not statistically significant. The patients with more than 3 diseases and over 60 years of age were more adherent to prescribed therapy (OR = 1.4416, *p* = 0.4414 and OR = 1.2647, *p* = 0.6153, respectively) (Table 5). For this reason, it should be concluded that no statistical reason exists to claim that MA level differs between different men and women, age groups, number of prescribed medicines, and number of diagnosed diseases. A significant related factor for MA was years lived with diabetes (OR = 3.039, 95% CI 1.1436–8.0759, *p* = 0.0258). The longer the disease duration, the more the odds for high MA level increased (Table 5).

## 4. Discussion

Diabetes is a chronic disorder which should be controlled by a diet, exercise, and pharmacological therapies to achieve glycemic control and prevent complications. Medication adherence is of exceptional importance in influencing diabetes and a positive outcome for the patient. Noncompliance with the therapeutic regime is responsible for complications and increased mortality. Higher adherence to therapy may be observed relative to disease duration. As the duration of illness increases, the proportion of higher adherents increases. Various studies have shown that nonadherence leads to increased direct and indirect costs [16].

Many factors could lead to lack of adherence: misperception of treatment benefits, a complex treatment scheme, and adverse events [17]. Focusing on adherence levels, it is critical to avoid future complications and achieve desired outcomes at the lowest possible cost. Patient adherence could be improved by various approaches such as patient involvement, active collaboration between healthcare professionals and patients, etc. [18]. Effective patient care could be achieved through education, supervision, and simplified therapeutic regimens. A reduction in self-control on the part of patients is necessary. Clinicians need to improve their prescribing and patient counseling to increase adherence [19]. Patients with lower adherence had more frequent hospitalizations and longer hospital stays than those with higher adherence [20].

Research on the level of medication adherence and related determinants among patients with diabetes is crucial. The results from the current study showed that the disease affects men and women equally (48 vs. 45), with 98.92% of them suffering from type 2 diabetes. The majority of patients were over 50 years old (90.33%). The current study showed that a greater proportion of patients were on biguanide therapy for diabetes (58 vs. 35) and were using more than 1 diabetes medication. Regarding accompanying diseases, the most common was hypertension (67.89%). No significant associations between sociodemographic characteristics (sex, age, living status, and diabetes duration) and adherence were identified. Data from other studies also showed a lack of association between these indicators and medication adherence. However, the current study affirmed that only a longer disease duration is related to a higher level of MA (OR = 3.039, *p* = 0.0258). There are controversial findings about duration of diabetes and the level of adherence. Gelaw et al. [21] revealed a statistically significant correlation between these two factors, as 82.07% of observed patients with a duration of diabetes ≤ 5 years were more likely to adhere. Other studies [22,23] revealed that patients treated for diabetes for more than 5 years were high-adherent, which is similar to our findings. This could be explained by the fact that these patients are better educated about their disease and possible complications, are more aware of their own condition, have more and probably stronger relations with their healthcare providers considering longer treatment, might have a better understanding of their medicines, and are more motivated to use the prescribed medications as they are prescribed by their physician.

Other studies found that male patients tended to consume alcohol and smoke more than female patients. This may be because social interactions between men are more likely to involve tobacco and alcohol, and because men are more likely to perceive smoking and alcohol consumption as desirable male behaviors [24]. Alcohol use is a barrier to management of medication adherence. Excessive alcohol consumption negatively impacts diabetes self-care and the course of diabetes [25]. However, our data do not provide any evidence for correlation between the level of adherence and alcohol consumption, probably due to the small sample size. Only a correlation between QoL scores and alcohol consumption was revealed, showing that smokers and patients drinking alcohol have better QoL scores than nonsmokers and those who did not consume alcohol. Our results are in contrast with other studies finding that the probability for higher quality of life is lower among smokers in comparison with nonsmokers [26]. Other studies concluded that people with low QoL and depression have higher odds to start smoking and lower odds to stop smoking [27]. The conflicting results are indicative of the necessity of conducting further, wider study among Bulgarian patients with diabetes.

Limitations of our study include the lack of analysis of the influence of factors such as levels of glycosylated hemoglobin (HbA1c), body mass index, and others. However, various studies provide evidence of this [28]. Another strong limitation is the limited number of patients observed. In the study, patients already diagnosed with diabetes from only one GP practice were examined. Given that there may be other undiagnosed diabetics, it would be beneficial to examine this group as well and conduct a comparison between the two groups. Many studies show a high number of patients with undiagnosed diabetes. We also observed that comorbidities play a role in patient adherence rates. Among the observed cohort of diabetic patients, the proportion of high-adherent patients diagnosed with several concomitant diseases exceeded the proportion of low- and middle-adherent patients. Other studies also found that medication adherence was higher among patients “with” in comparison with those “without” concomitant diseases, probably due to their increased concerns about worsening in their condition [29,30]. The odds of medication adherence among patients with chronic conditions taking four medicines are higher than those taking one medicine [30]. Indicators influencing adherence include the following: the high number of drugs that patients take, complex therapeutic regimens, and a large number of accompanying diseases [31].

Few patients in the studied GP practice had weak adherence—only 2% of the observed cohort. The patients diagnosed with more comorbidities have better medication adherence, which could be explained with stricter follow-up of the condition and patients’ awareness about their own health. Factors related to the physician’s and patients’ behavior and attitude towards medication adherence issues influencing the high level of medication adherence should be investigated in further studies. The study raises questions about medication adherence in patients with diabetes and their treatment. The need for more documentation for patients with diabetes is strongly emphasized. There are not many similar studies in Bulgaria. This study may be a stepping stone for larger studies analyzing the influence and specifics of GP practice on the monitoring and improving of MA among diabetic patients from ambulatory practice.

## 5. Conclusions

The number of nonadherent diabetic ambulatory patients observed in GP practices in Bulgaria is low, which might be proof of patients’ concern about their own health and understanding about the importance of prescribed therapy. This hypothesis should be further investigated; thus, we are planning to conduct a subsequent study. A comprehensive study including more patients is needed to confirm the results and to investigate the predicting factors for a high level of MA.

## Figures and Tables

**Table 1:** Demographic data of the observed patients (*n* = 93).

	Subset	Distribution N (%)	*p* Value **
Gender	Women	45 (48.39%)	*p* = 0.75
	Men	48 (51.61%)	
Age group (years)	Up to 30 years	1(1.07%)	
	30–40 years	-	
	41–50 years	8(8.6%)	
	50–60 years	19(20.43%)	
	61–70 years	20(21.61%)	
	over 70 years	45(48.39%)	
	over 50+ years	84(90.33%)	
Professional status	Working	39 (41.94%)	*p* = 0.12
	Retirees	54 (58.06%)	
Type of diabetes	Type 1	1	
	Type 2	92 (98.92%)	
Duration of the disease	Up to 5 years	36 (38.71%)	
	6–10 years	24 (25.81%)	
	11–15 years	20 (20.43%)	
	16–20 years	10 (10.75%)	
	Over 21 years	4 (4.3%)	
Smokers	Women	13 (28.88%)	*p* = 0.19
	Men	31 (64.58%)	
Alcohol consumption *	Women	13 (28.88%)	*p* = 0.22
	Men	30 (62.50%)	
Control of high blood pressure levels	Women	36 (80%)	*p* = 0.92
	Men	37 (77.08%)	
Concomitant diseases	Women	45 (48.39%)	*p* = 0.76
	Men	48 (51.61%)	

* Alcohol consumption is related to any amount of drinking alcohol during the day. ** No statistically significant difference was observed.

**Table 2:** Pharmacotherapy for the concomitant diseases.

Pharmacological Group	Number of Patients	INN *
**Antihypertensives**		
ACE inhibitors *	9	Perindopril Ramipril
AT1 blockers *	14	Valsartan Olmesartan
Agents affecting the sympathoadrenal system	66	Moxonidine Bisoprolol
Calcium antagonists	23	Felodipine Nifedipine Verapamil
Antihypertensive diuretics	16	Indapamide Spironolactone Furosemide
Combined drugs	44	Enalapril/hydrochlorthiazide Valsartan/hydrochlorthiazide
**Antihyperlipidemic**		
Statin	30	Rosuvastatin Atorvastatin
**Cardiac therapy**		
Antiarrhythmic	1	Propafenone hydrochloride
Antianginal	4	Molsidomine
**Anticoagulants**		
K1 antagonists	1	Acenocoumarol
Direct inhibitors of thrombin	1	Dabigatran
Direct inhibitors of Xa factor	9	Apixaban
Antiplatelet agents	7	Clopidogrel
**Peripheral vasodilators**		
Alpha adrenergic blockers	6	Tamsulosin
Phosphodiesterase inhibitors	5	Pentoxifylline
Cerebral vasodilators	8	Vinpocetine
Combined drugs for benign prostatic hyperplasia	3	Solifenacin succinate/tamsulosin Dutasteride/tamsulosin hydrochloride
Drugs affecting Polyneuropathies	14	Thioctic acid
**Thyroid treatment**		
Thyroid preparations	10	Levothyroxine sodium
**Antiparkinsonian**		
Affecting dopaminergic neurotransmission	1	Pramipexole
Drugs affecting central cholinergic neurotransmission	1	Biperiden hydrochloride
Combined antiparkinsonian drugs	1	Levodopa/benserazide
Antiepileptics	1	Gabapentin
Antipsychotics	4	Piracetam
**Antiglaucoma**		
Prostaglandin analogues	6	Latanoprost
Combined drugs	3	Dorzolamide/timolol

* ACEI—angiotensin-converting enzyme inhibitors; AT1—angiotensin II receptor blocker; INN—international non-proprietary name.

**Table 3:** EQ-5D5L and VAS questionnaires.

	Gender		
	Male	Female	*p* Value
EQ-5D-5L	0.65 ± 0.31	0.47 ± 0.31	0.063
	**Smoking status**		
	Smokers (*n* = 44)	Nonsmokers (*n* = 49)	*p* Value
EQ-5D-5L	0.62 ± 0.31	0.51 ± 0.32	*p* = 0.09
VAS	72.77 ± 14.3	63.16 ± 20.45	*p* = 0.01 *
	**Alcohol consumption**		
	Yes (*n* = 41)	No (*n* = 52)	*p* Value
EQ-5D-5L	0.648 ± 0.31	0.49 ± 0.32	*p* = 0.017 *
VAS	72.54 ± 15.98	63.91 ± 19.34	*p* = 0.02 *
	**Medication adherence**		
	High level	Medium level	*p* Value
EQ-5D-5L	0.533 ± 0.32	0.602 ± 0.32	*p* = 0.33

* Statistically significant result: *p* < 0.05.

**Table 4:** Medication adherence and related factors.

Medication Adherence (MA)	Weak	Average	High	Number
Total number [%] *	2 [2.15%]	31 [33.33%]	60 [64.52]	93
**MA by gender**				
Men [%]	0 [0]	20 [41.67%]	28 [58.33%]	48 [51.61%]
Women [%]	2 [4.44%]	11 [24.44%]	32 [71.11%]	45 [48.39%]
**MA vs. professional status**				
Working [%]	1 [2.56%]	17 [43.59%]	21 [53.85%]	39 [41.93%]
Retired [%]	1 [1.85%]	14 [25.93%]	39 [22.72%]	54 [58.06%]
**MA versus number of therapies**				
Monotherapy [%]	1 [1.97%]	19 [33.93%]	36 [29.64%]	56 [60.22%]
Two therapies [%]	1 [3.13%]	10 [31.25%]	21 [65.63%]	32 [34.4%]
Three therapies [%]	0 [0%]	2 [40%]	3 [60%]	5 [5.38%]
**Duration of the disease**				
Up to 5 years [%]	2 [5.71%]	15 [42.86%]	18 [51.43%]	35 [37.63%]
6–10 years [%]	0	9 [37.50%]	15 [62.50%]	24 [25.81%]
11–15 years [%]	0	4 [20.00%]	16 [80%]	20 [21.51%]
16–20 years [%]	0	3 [30.00%]	7 [70%]	10 [10.75%]
over 21 years [%]	0	0	4 [100%]	4 [4.3%]
**Number of comorbidities**				
One concomitant disease [%]	2 [7.14%]	7 [25%]	19 [67.86%]	28 [30.10%]
Two additional concomitant diseases [%]	0	14 [41.18%]	20 [58.82%]	34 [36.55%]
Three additional concomitant diseases [%]	0	5 [25%]	15 [75%]	20 [21.50%]
Four additional concomitant diseases [%]	0	3 [37.5%]	5 [62.50%]	8 [8.6%]
Five additional concomitant diseases [%]	1 [33.33%]	1 [33.33%]	1 [33.33%]	3 [3.22%]
**Costs for treatment**				
Paid by patients	19.52 ± 2.1	32.89 ± 23.85	37.31 ± 25.56	
**Type of therapy**				
Insulin secretagogues	0	2 [16.67%]	10 [83.3%]	12
metformin	0	13 [43.3%]	17 [56.67%]	30
Insulin secretagogues + metformin	1 [8.3%]	5 [41.6%]	6 [50%]	12

* The relative share of each absolute number is presented in brackets in percentages [%].

**Table 5:** Logistic regression results.

Determinant	High Level of MA	Medium and Low Level of MA	OR, 95% CI, *p*
Gender			OR = 1.7582
Male	28	20	95% CI 0.7418–4.1667
Female	32	13	*p* = 0.2
Polypharmacy			OR = 1.3176
≥5 medicines	35	17	95% CI 0.5609–3.0955
<5 medicines	25	16	*p* = 0.5267
Multimorbidity			OR = 1.4416
≥3 diseases	22	9	95% CI 0.5681–3.6578
<3 diseases	39	23	*p* = 0.4414
Age			OR = 1.2647
<60 years	17	11	95% CI 0.5060–3.1610
≥60 years	43	22	*p* = 0.6153
Years lived with diabetes			OR = 3.039
<10 years	33	26	95% CI 1.1436–8.0759
≥10 years	27	7	*p* = 0.0258 *

* Statistically significant result: *p* < 0.05; MA—medication adherence.

## Data Availability

The data could be provided upon request.
